# Low-dose aspirin and anaemia risk in pregnancy: A scoping review with emphasis on low- and middle-income contexts

**DOI:** 10.4102/safp.v68i1.6195

**Published:** 2026-04-08

**Authors:** Nokwethemba M. Ngcobo, Vinogrin Dorsamy, Chauntelle Bagwandeen

**Affiliations:** 1 School of Laboratory Medicine and Medical Science, College of Health Sciences, University of KwaZulu-Natal, Durban, South Africa; 2 Department of Public Health Medicine, School of Nursing and Public Health, College of Health Sciences, University of KwaZulu-Natal, Durban, South Africa

**Keywords:** low-dose aspirin, haemoglobin, anaemia, preeclampsia, hypertensive disorders of pregnancy, gastrointestinal bleeding, obstetric haemorrhage

## Abstract

**Background:**

Anaemia remains a major public health concern in pregnancy and is associated with adverse maternal and perinatal outcomes. Low-dose aspirin (LDA) is widely prescribed to reduce the risk of preeclampsia (PE), yet its effects on haemoglobin (Hb) levels and anaemia risk are poorly characterised.

**Methods:**

This scoping review mapped available evidence on the association between LDA use and Hb concentration or anaemia outcomes in pregnant women. The review followed Joanna Briggs Institute methodology and was reported in accordance with PRISMA-ScR. PubMed, Google Scholar, Scopus, Web of Science and the Cochrane Library were searched from inception to December 2025. Eligible studies included pregnant women prescribed LDA, a comparator group and reported Hb- or anaemia-related outcomes, with particular focus on low- and middle-income country (LMIC) settings. Studies were screened, and data were extracted using a standardised charting tool by one reviewer and independently checked by a second reviewer. Evidence was analysed descriptively using a two-stage approach.

**Results:**

Thirteen studies met the inclusion criteria, comprising one case report with a narrative review, four observational cohort studies, four systematic reviews, two randomised controlled trials (RCTs) and two secondary analyses of RCTs. Most studies evaluated LDA for PE prevention, while only two pregnancy-specific studies directly assessed Hb outcomes. Bleeding-related haematologic endpoints were variably reported, but no study evaluated anaemia incidence.

**Conclusion:**

Evidence on the relationship between LDA use and anaemia in pregnancy remains limited and inconclusive. Available studies lack standardised haematologic endpoints, and LMIC-specific data are sparse.

**Contribution:**

This review highlights critical evidence gaps and underscores the need for well-designed prospective studies incorporating standardised anaemia-related outcomes in pregnant populations, particularly in LMIC contexts.

## Introduction

Low-dose aspirin (LDA) is increasingly being recognised for its role in the prevention of preeclampsia (PE), a hypertensive disorder of pregnancy (HDP). Globally, HDPs continue to cast a long and persistent shadow over maternal and perinatal health, accounting for a significant proportion of obstetric morbidity and mortality.^1,2^ Low- and middle-income countries (LMICs) bear the heaviest brunt of this burden, since health systems often lack capacity, and late antenatal booking remains common.^3,4^ South Africa (SA) is no exception, for nearly one in five maternal deaths is attributed to HDP.^5^ In recent years, the use of LDA has become a more widely adopted strategy to interrupt this trajectory, offering a simple yet powerful intervention to prevent PE if prescribed at the appropriate gestational age.^6,7^ South African guidelines recommend LDA for women at high risk of PE, with doses ranging from 75 mg to 150 mg daily, advising initiation around 12 weeks of gestation and continuation until approximately 36 weeks.^8^

Preeclampsia is a multifaceted syndrome characterised by new-onset hypertension with organ dysfunction after 20 weeks of gestation. Its origins lie in defective placentation, poor spiral artery remodelling and systemic endothelial dysfunction – pathways that collectively compromise maternal and foetal well-being.^9,10^ Low-dose aspirin works primarily by irreversibly inhibiting platelet cyclooxygenase-1 (COX-1), suppressing thromboxane A_2_ (TXA2) – a potent vasoconstrictor and pro-thrombotic agent – thereby restoring the delicate vascular balance needed to ensure healthy placental perfusion.^11,12^

Alongside the burden of HDP is the global crisis of anaemia in pregnancy, which further erodes quality maternal health in LMICs. Defined by the World Health Organization (WHO) as haemoglobin (Hb) below 11 g/dL, anaemia affects over 35% of pregnant women globally.^13^ In many low-resource settings, it is driven by a complex web of causation, consisting of nutritional deficiencies, chronic inflammation and structural barriers to care.^14,15^ Anaemia increases the risk of preterm birth, low birth weight and perinatal mortality, and its consequences – though often less visible than those of PE – are no less profound.^16,17^

Haemoglobin concentrations in pregnancy follow a non-linear pattern of risk.^18^ Both low and high levels are associated with adverse outcomes: anaemia reduces oxygen delivery to the foetus, while elevated Hb may signal inadequate plasma volume expansion or iron overload, increasing the risk of placental dysfunction and hypertension.^19,20^

These conditions – PE and anaemia – do not operate in isolation. Instead, they often coexist in a physiological tug-of-war. Preeclampsia can suppress red cell production through systemic inflammation and oxidative stress, while anaemia exacerbates placental hypoxia and maternal cardiovascular strain.^21,22,23^ This is particularly relevant in LMICs, where coexisting burdens such as human immunodeficiency virus (HIV), tuberculosis, malaria and obesity intersect with sub-optimal maternal nutrition, further complicating management.

As LDA becomes embedded into global antenatal guidelines, the yet-to-be-resolved question is whether it might influence Hb levels, especially in anaemia-prone populations. While LDA’s vascular benefits are well established, emerging concerns include its potential to cause subclinical gastrointestinal bleeding, interfere with iron absorption or modulate inflammatory pathways that affect erythropoiesis.

## Objectives

The specific objectives of this review were to:

- Examine existing evidence on the association between LDA use and Hb or anaemia outcomes in pregnant women.
- Describe underlying biological mechanisms through which LDA may influence iron metabolism, erythropoiesis and red-cell indices.
- Identify contextual factors and evidence gaps relevant to LMIC settings, where the dual burden of hypertensive disorders and anaemia remains high.

The review question was conceptualised using the Population–Intervention–Comparator and Outcome (PICO) framework:

- Population: Pregnant women, particularly in LMICs
- Intervention: Women prescribed LDA.
- Comparator: Placebo or no treatment.
- Outcome: Changes in Hb, incidence of anaemia, bleeding or blood loss and haemorrhage.

## Research methods and design

This scoping review followed the Joanna Briggs Institute (JBI) methodological framework, which guided the development of the review objectives, eligibility criteria, search strategy, evidence selection, data charting and synthesis.^24^ Reporting was aligned with the Preferred Reporting Items for Systematic Reviews and Meta-Analyses extension for Scoping Reviews (PRISMA-ScR) guideline to ensure transparency and completeness.^25^ Scoping reviews, as outlined in the JBI Manual for Evidence Synthesis, aim to map the breadth and nature of available evidence rather than evaluate intervention effects or generate pooled estimates. This approach was appropriate for the present review, given the heterogeneity of study designs, outcomes and mechanistic evidence on LDA and anaemia-related endpoints during pregnancy.

### Eligibility criteria

Studies were eligible for inclusion if they:

- Included pregnant women at any gestational age.
- Reported exposure to LDA prescribed during pregnancy (typically ≤ 165 mg/day).
- Included a comparator group, defined as placebo, standard antenatal care or no aspirin use.
- Reported at least one of the following outcomes; namely changes in Hb levels; maternal bleeding, blood loss or haemorrhage; and/or incidence or prevalence of anaemia.
- Included populations from LMICs, with some studies from high-income countries (HICs) where relevant to contextualise the evidence.
- Employed any study design, including randomised controlled trials (RCTs), cohort studies, case–control studies, observational studies, systematic reviews, case reports, narrative or literature reviews, dissertations, theses and international guidelines.

Studies were excluded if they were editorials, commentaries, opinion pieces or animal studies.

There were no language restrictions. Where necessary, non-English articles were translated using Google Translate or professional translation services to maximise evidence capture, consistent with scoping review methodology.

### Information sources

A comprehensive literature search was conducted across five databases in PubMed, Google Scholar, Scopus, Web of Science and the Cochrane Library covering all records from inception to December 2025. In addition to electronic databases, we also search the reference lists of included articles (backward citation chaining) and use forward citation tracking via Google Scholar to identify additional relevant studies.

### Search strategy

The search terms and synonyms were developed using Medical Subject Headings (MeSH) and piloted in PubMed. The final PubMed search string was:

((“Aspirin” [MeSH Terms] OR “low-dose aspirin” OR “acetylsalicylic acid”) AND (“Anemia” [MeSH Terms] OR “haemoglobin” OR “hemoglobin” OR “blood iron levels” OR “iron deficiency” [MeSH Terms]) AND (“Pregnancy” [MeSH Terms] OR “pregnant women” OR “maternal” OR “pregnancy complications” [MeSH Terms] OR “hypertensive disorder of pregnancy” [MeSH Terms] OR “preeclampsia” [MeSH Terms] OR “pregnancy outcomes” [MeSH Terms] OR “obstetric hemorrhage” [MeSH Terms] OR “postpartum hemorrhage” [MeSH Terms])). This search strategy was adapted for all other databases. The search was peer-reviewed by an information specialist according to the Peer Review of Electronic Search Strategies (PRESS) checklist to optimise sensitivity and precision.^26^ To ensure comprehensive coverage, grey literature sources were included, such as ProQuest dissertations, theses and international guidelines or policies where published studies were limited.

### Evidence screening and selection

All retrieved citations were imported into Zotero (v5.0.81) for reference management and duplicate removal using its built-in detection tool and manual verification. Title and abstract screening were performed using Rayyan software by two independent reviewers (Nokwethemba M. Ngcobo and Vinogrin Dorsamy) according to predefined inclusion and exclusion criteria.^27^ Any disagreements arising during the screening process were addressed through mutual discussion or, if necessary, by seeking input from a third reviewer (Chauntelle Bagwandeen) to reach consensus. A calibration exercise was conducted prior to screening to ensure consistency and shared understanding of the eligibility criteria. Full-text articles deemed potentially relevant were retrieved and assessed for eligibility by two independent reviewers, and reasons for exclusion at this stage were recorded to maintain transparency.

### Charting the data

Data were charted using a standardised extraction table developed in Microsoft Excel, guided by the JBI and PRISMA-ScR principles. Extracted information included citation details (author and year of publication), study design, country and population characteristics. Details of the intervention or exposure (LDA dose, timing and indication), comparator (if applicable) and outcomes measured – such as Hb levels, anaemia prevalence, ferritin concentration and related haematological indices – were recorded. Information on the indication for LDA use (e.g. PE or HDP) was extracted where reported. Contextual variables, including healthcare setting, comorbidities, nutritional status and other relevant sociodemographic factors, were also documented. Data extraction was conducted by one reviewer and independently checked by a second reviewer for accuracy and completeness. Prior to full charting, the data extraction tool was piloted on a subset of studies by two independent reviewers to ensure clarity and consistency.

### Analysis of evidence

Evidence was analysed using the two stages of the Arksey and O’Malley framework.^28^ Firstly, the study selection process was mapped using a PRISMA-ScR flow diagram to provide an overview of the identification, screening and inclusion of sources of evidence. Secondly, a descriptive summary of the included studies was generated and presented in tabular form, detailing key study characteristics such as study design, setting, sample size, LDA dose and timing and reported outcome measures.

### Ethical consideration

This review is part of a larger study investigating the impact of LDA on Hb levels during pregnancy, which obtained ethical approval from the University of KwaZulu-Natal (UKZN) Biomedical Research Ethics Committee (BREC reference BREC/00007156/2024).

## Results

**FIGURE 1:**
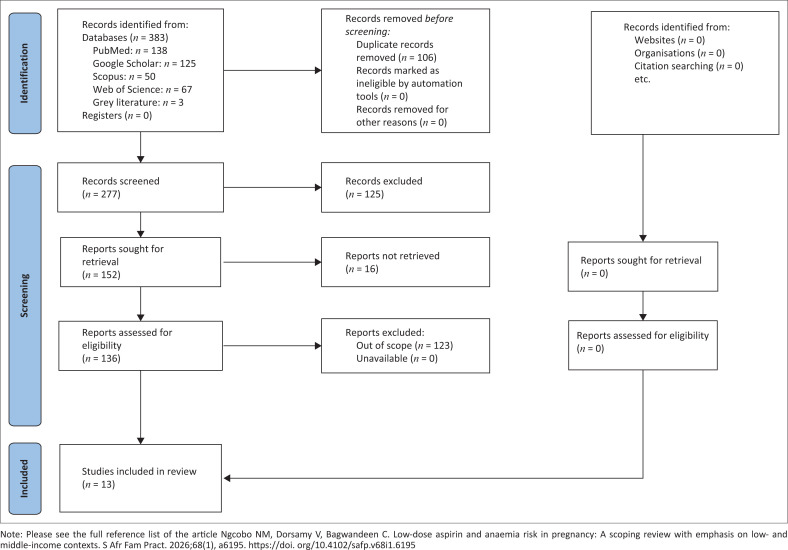
Preferred Reporting Items for Systematic Reviews and Meta-Analysis for Scoping Review (PRISMA-ScR) flow chart for selection of studies.^29^

The search identified a heterogeneous body of literature, including RCTs, observational studies and systematic reviews relevant to LDA and haematologic outcomes. After screening 383 records and reviewing full texts, 13 studies met the eligibility criteria and were included in the final analysis. A PRISMA-ScR flow diagram summarises the selection process (Figure 1).^29^

The included studies varied in design, population, indication for LDA use, dosage (typically 60 mg/day – 165 mg/day), outcomes reported and contextual relevance to LMICs. As several individual studies addressed more than one review objective, results are presented descriptively by mapping the available evidence across key outcome domains – namely Hb changes, bleeding-related outcomes and anaemia incidence or prevalence. Within each outcome domain, the number and type of studies, reported findings and identified evidence gaps are summarised in relation to the review objectives.

### Outcome 1: Changes in haemoglobin levels

Two studies reported on changes in maternal Hb levels. Both were secondary analyses of RCTs. Jessani et al., a secondary analysis of the ASPIRIN trial, enrolled nulliparous pregnant women from multiple LMICs between 6 and 13 weeks of gestation.^30^ Ngcobo et al. included pregnant women of African ancestry (*n* = 249) with singleton, normotensive pregnancies attending a regional hospital in Durban, South Africa, enrolled between 12 and 20 weeks of gestation.^31^

#### Low-dose aspirin dosage and timing

In the ASPIRIN trial secondary analysis, participants received 81 mg of LDA daily, initiated in the first trimester (6–13 weeks) and continued throughout pregnancy. Haemoglobin concentration was measured in the first trimester and again at 26–30 weeks of gestation. In contrast, Ngcobo et al.^31^ evaluated 162 mg of LDA daily, initiated between 12 and 20 weeks of gestation and continued until delivery. Haemoglobin change was assessed from enrolment to delivery.

#### Outcomes reported

Jessani et al.^30^ reported:

- Maternal Hb concentration at two time points (first trimester and 26–30 weeks of gestation).
- Comparisons of mean Hb levels between aspirin and placebo groups at each time point.

Ngcobo et al.^31^ reported:

- Change in maternal Hb concentration from enrolment to delivery.
- Associations between the magnitude of Hb decline and the risk of hypertensive disorders of pregnancy (HDP).

#### Key findings

Jessani et al.^30^ found no significant differences in mean Hb concentrations between the aspirin and placebo groups at either the first-trimester measurement or at 26–30 weeks of gestation. Ngcobo et al.^31^ reported smaller declines in Hb from enrolment to delivery amongst women receiving 162 mg of LDA compared with controls. Additionally, larger declines in Hb were associated with an increased risk of HDP.

#### Gaps identified

The following gaps were evident:

- Only two pregnancy-specific studies directly evaluated LDA in relation to Hb or anaemia-related outcomes. Haemoglobin changes were a primary outcome in Ngcobo et al.,^31^ and Jessani et al.^30^ reported Hb concentration as a secondary outcome.
- Both studies were secondary analyses of RCTs; no primary trials were specifically designed with Hb or anaemia as primary endpoints in pregnancy.
- Differences in aspirin dose (81 mg vs 162 mg) and timing of Hb assessment limit comparability across studies.
- No included study systematically assessed iron status (e.g. ferritin, transferrin saturation), dietary iron intake or biological pathways related to iron metabolism, erythropoiesis or red-cell indices in relation to LDA exposure during pregnancy.
- Although Ngcobo et al.^31^ adjusted for potential confounders such as HIV status, no association between HIV and changes in Hb was observed.
- Evidence is limited to specific populations (nulliparous women in a multi-country LMIC trial and women of African ancestry from a single South African centre), restricting generalisability to broader LMIC settings.

### Outcome 2: Maternal bleeding, blood loss or haemorrhage

Obstetric bleeding represents a potential pathway through which LDA may influence maternal Hb and anaemia risk; therefore, studies reporting maternal bleeding outcomes in relation to LDA use during pregnancy were examined. Eleven heterogeneous sources of evidence were identified, including case reports, observational cohort studies, two multicentre RCTs and systematic reviews. A single case report with a narrative literature review by Plancha et al. described a pregnant woman using LDA (75 mg/day – 150 mg/day) for PE prevention who developed life-threatening upper gastrointestinal bleeding, illustrating that serious haemorrhagic events can occur, although rarely, during LDA exposure.^32^ At the population level, Hastie et al. conducted a large registry-based cohort study in Sweden (approximately 315 000 pregnancies; 1.3% exposed to aspirin 75 mg/day – 160 mg/day) and reported an increased risk of bleeding during labour among aspirin-exposed pregnancies; anaemia outcomes were not assessed.^33^ Similarly, White et al., in an observational cohort of women prescribed 81 mg LDA for early PE prophylaxis, reported a higher risk of postpartum bleeding compared with non-use.^34^ In a multicentre propensity score–based cohort study, Souter et al. reported increased odds of placental abruption (OR 1.44) and postpartum haemorrhage (OR 1.21) among pregnancies exposed to LDA.^35^

In contrast, high-quality RCT evidence demonstrates neutral bleeding safety profiles. Rolnik et al. conducted a multicentre double-blind RCT of 1776 high-risk pregnancies across 13 countries, randomising women at 11–14 weeks to 150 mg nightly aspirin until 36 weeks versus placebo and found postpartum haemorrhage rates of 4.4% (35/798) in the aspirin group versus 4.0% (32/798) in placebo (RR 1.09, 95% CI 0.69–1.73) with no significant increase in placental abruption (0.3% vs 0.1%).^7^ Similarly, Lin et al. reported findings from the largest Chinese multicentre RCT (*n* = 3236 high-risk women enrolled 12–20 weeks across 13 tertiary hospitals), comparing 100 mg daily aspirin until 34 weeks versus standard care, which showed no significant differences in postpartum haemorrhage or other bleeding complications between groups as safety endpoints.^36^

Other observational studies and systematic reviews provide mixed findings. Zhang and Wang, in a retrospective cohort of high-risk pregnant women, found no significant difference in postpartum haemorrhage rates between aspirin-exposed and non-exposed groups.^37^ Jiang et al. conducted a systematic review of 21 studies reporting postpartum haemorrhage (*n* = 373 926) and seven reporting blood loss (*n* = 10 163) among women receiving 60 mg/day – 150 mg/day aspirin and found a modestly increased risk of postpartum haemorrhage (OR 1.20, 95% CI 1.07–1.34) and greater blood loss in LDA users.^38^ However, Roberge et al., in a systematic review of 45 RCTs (*n* = 20 909) in which LDA (≥ 100 mg/day) was initiated at or before 16 weeks of gestation, found no significant increase in placental abruption or antepartum haemorrhage.^39^ Similarly, Henderson et al. reported no significant increase in haemorrhagic complications in high-risk pregnancies treated with 60 mg/day – 150 mg/day aspirin initiated at ≤ 16 weeks of gestation, while Duley et al., in a systematic review of 60 RCTs, reported a small increase in minor bleeding events but no clinically important excess in major haemorrhage or blood loss.^40,41^

Overall, the available evidence primarily addresses obstetric bleeding outcomes – such as intrapartum bleeding, postpartum haemorrhage, placental abruption and rare gastrointestinal haemorrhage – without directly examining Hb or anaemia outcomes in relation to bleeding among pregnant women using LDA. The evidence base is methodologically diverse and occasionally discordant, with several observational studies and one systematic review suggesting modest increases in postpartum haemorrhage or blood loss, whereas two RCTs generally do not demonstrate a clinically meaningful increase in haemorrhagic events.^7,33,34,35,36,37,38^ Notably, few studies originate from LMIC settings with a high background prevalence of anaemia, and none explicitly link LDA-related bleeding to subsequent maternal Hb or anaemia outcomes during pregnancy or the postpartum period.^30,31,36,37^

### Outcome 3: Incidence or prevalence of anaemia

None of the included studies directly reported the incidence or prevalence of anaemia following LDA use during pregnancy. Although several studies evaluated related outcomes, such as Hb concentration or obstetric bleeding, anaemia was not measured or analysed as a primary or secondary endpoint in any study. Consequently, no quantitative or qualitative synthesis could be performed for this outcome.

As shown in Table 1, LDA is well established as an effective intervention for preventing PE, yet few studies have directly examined its impact on Hb levels or anaemia risk in pregnancy. One study reported no meaningful difference in Hb trajectories between aspirin and placebo groups, whereas another observed smaller declines in Hb among women receiving LDA, with larger Hb reductions associated with increased HDP risk.^30,31^ Several large RCTs and systematic reviews have evaluated LDA for PE prevention in high-risk pregnancies; however, anaemia-related outcomes were infrequently reported.^7,36,41^

**TABLE 1:** Characteristics of 15 studies investigating low-dose aspirin and anaemia-related or haematological outcomes.

Author	Study design and population	LDA dosage and timing	Key findings or outcomes
Jessani et al.^30^	Secondary analysis of ASPIRIN Trial (RCT); nulliparous women at 6–13 weeks of gestation from LMICs.	81 mg daily; Hb measured at first trimester and 26–30 weeks	No significant difference in Hb levels between the aspirin and placebo groups at both time points.
Ngcobo et al.^31^	Secondary analysis of RCT; pregnant women of African ancestry (*n* = 249) at a regional hospital in Durban, South Africa, normotensive with singleton pregnancies at 12–20 weeks of gestation.	162 mg daily; initiated between 12–20 weeks until birth, with Hb measured from enrolment to delivery	LDA was associated with smaller Hb declines vs controls; greater Hb drops correlated with higher HDP risk, suggesting haematological benefit that could mitigate anaemia progression and Hb change as a potential early HDP indicator.
Plancha et al.^32^	Case report and literature review; pregnant woman using LDA for PE prevention.	75 mg/day – 150 mg/day	Reported life-threatening upper gastrointestinal bleeding; serious bleeding is rare but possible during LDA use.
Hastie et al.^33^	Population-based cohort (Sweden, 2013–2017); 4088/315 000 pregnancies (1.3%) with aspirin exposure	75 mg/day – 160 mg/day	LDA associated with an increased risk of bleeding during labour. Anaemia-related outcomes were not reported.
Jiang et al.^38^	Systematic review and meta-analysis; including 21 studies (including RCTs and cohort studies, total *n* = 373 926 women) on postpartum haemorrhage and 7 studies (*n* = 10 163) on blood loss, involving pregnant women using LDA for various indications	60 mg/day – 150 mg/day during pregnancy	LDA increased the risk of postpartum haemorrhage (OR 1.20, 95% CI 1.07–1.34) and blood loss.
White et al.^34^	Observational cohort of pregnant women	81 mg daily; early initiation for PE prevention	LDA use may be associated with increased postpartum bleeding risk.
Zhang and Wang^37^	Retrospective cohort study of high-risk pregnant women for PE.	Not specified	No significant difference in postpartum haemorrhage between aspirin and non-aspirin groups (*p* > 0.05).
Roberge et al.^39^	Systematic review of 45 (*n* = 20 909) RCTs on LDA for PE prevention.	Initiated ≤ 16 weeks; ≥ 100 mg/day	No significant increase in placental abruption or antepartum haemorrhage with LDA.
Souter et al.^35^	Multicentre observational cohort using propensity score analysis; pregnant women recommended LDA	Not specified	Increased risk of placental abruption (OR 1.44) and postpartum haemorrhage (OR 1.21).
Duley et al.^41^	Systematic review and meta-analysis of 60 RCT; pregnant women at increased risk for PE.	Not specified	Reported mild bleeding risk with no clinically significant blood loss; anaemia-related outcomes were not reported.
Henderson et al.^40^	Systematic review and meta-analysis; high-risk pregnancies for PE.	60 mg/day – 150 mg/day, ≤ 16 weeks of gestation	LDA reduced perinatal mortality and foetal growth restriction; no significant increase in haemorrhage.
Rolnik et al.^7^	Multicentre double-blind RCT; 1776 pregnancies at high risk for preterm PE (< 37 weeks), recruited at 11–14 weeks of gestation across 13 countries.	150 mg nightly aspirin from 1 to –14 weeks of gestation until 36 weeks or delivery vs placebo	Postpartum haemorrhage 35/798 (4.4%) aspirin vs 32/798 (4.0%) placebo (RR 1.09, 95% CI 0.69–1.73, *p* = 0.71); placental abruption 2/798 (0.3%) vs 1/798 (0.1%) (no significant difference). No Hb concentrations or anaemia incidence reported.
Lin et al.^36^	Multicentre RCT; 3236 high-risk pregnant women for PE across 13 tertiary hospitals in China, enrolled 12–20 weeks of gestation.	100 mg daily aspirin from 12 to 20 weeks until 34 weeks of gestation vs standard care.	No PE reduction; postpartum haemorrhage and bleeding complications showed no significant group differences. No Hb or anaemia data reported.

Note: Please see the full reference list of the article Ngcobo NM, Dorsamy V, Bagwandeen C. Low-dose aspirin and anaemia risk in pregnancy: A scoping review with emphasis on low- and middle-income contexts. S Afr Fam Pract. 2026;68(1), a6195. https://doi.org/10.4102/safp.v68i1.6195.

RCT, randomised controlled trials; LMIC, low- and middle-income country; LDA, low-dose aspirin; HDP, hypertensive disorder of pregnancy; PE, preeclampsia; Hb, haemoglobin.

Observational studies and one systematic review indicated a possible association between LDA and increased risks of postpartum bleeding or haemorrhage.^33,34,38^ However, some large trials and other systematic reviews reported no clinically significant increase in obstetric haemorrhage.^7,39^

## Discussion

This scoping review mapped current evidence on the relationship between LDA and anaemia-related outcomes in pregnancy, with a particular attention to LMIC contexts. The overarching finding is that, despite widespread guideline recommendations for LDA to prevent PE and other HDP, its effects on Hb, iron status and anaemia classification remain poorly characterised.^6,42^ Most trials and observational studies were designed to evaluate LDA for PE prevention, with haematologic outcomes reported primarily as bleeding or haemorrhage endpoints rather than anaemia.^7,38,40^ While the direct relevance of bleeding in these studies to anaemia development remains uncertain, even mild or subclinical bleeding may increase anaemia risk among pregnant women in LMICs, where baseline anaemia prevalence is already high.^43^

Only two pregnancy-specific studies directly evaluated Hb outcomes in relation to LDA use, both as secondary analyses of RCTs.^30,31^ Notably, systematic reviews assessing LDA for PE or HDP prevention did not predefine Hb or anaemia as outcomes, underscoring the limited attention given to anaemia.^39,40,41^

Mechanistic evidence, derived from narrative reviews and in vitro pregnancy studies, suggests both potentially harmful and potentially protective pathways.^44,45,46^ Aspirin and its metabolites may bind iron and reduce its bioavailability, potentially impairing absorption and contributing to iron deficiency anaemia (IDA) in susceptible individuals.^44,45^ Conversely, LDA can attenuate systemic inflammation, including reductions in interleukin 6 (IL-6), a key regulator of hepcidin synthesis.^46^ Lower hepcidin levels could improve iron absorption and mobilisation from stores, thereby ameliorating anaemia of inflammation. At present, no included studies directly assessed LDA’s impact on hepcidin, iron indices and erythropoiesis in pregnant women, particularly in those with PE or coexisting infections. Consequently, the net haematologic effect of LDA in pregnancy remains uncertain. In LMICs, the interplay between LDA, anaemia and HDP is further shaped by contextual factors. Chronic infections such as HIV, tuberculosis and malaria cause persistent inflammatory states, elevate hepcidin and impair iron utilisation, predisposing women to anaemia of chronic disease.^47,48,49^ While Ngcobo et al.^31^ adjusted for potential confounders such as HIV status, no association between HIV and changes in Hb was observed. However, in malaria-endemic regions, infection modified LDA’s impact on pregnancy outcomes, including perinatal mortality.^50^ Nutritional deficiencies – particularly iron, folate and vitamin B12 – remain prevalent while rising obesity introduces a parallel burden of chronic low-grade inflammation and altered iron metabolism.^51,52^ Genetic conditions such as sickle cell trait, thalassaemia and glucose-6-phosphate dehydrogenase (G6PD) deficiency may further alter haematologic responses to iron supplementation.^53,54^ Structural and health system barriers, including late antenatal booking, limited diagnostic capacity, inconsistent availability of iron supplements or aspirin and fragmented continuity of care, may delay LDA initiation, hinder Hb monitoring and amplify anaemia risk.^55,56^

Taken together, these findings indicate that the safety profile of LDA with respect to anaemia cannot be assumed to be uniform across all populations. The balance between its vascular benefits and potential haematologic consequences is likely to be context dependent, influenced by baseline anaemia prevalence, infection burden, nutritional status and health-system factors. Addressing these gaps will require study designs that mirror real-world antenatal care in LMICs, incorporate anaemia-related endpoints and allow stratified analyses by nutritional status, infection and comorbidity profiles, genetic background and gestational age at LDA initiation. In parallel, include mechanistic assessments of iron metabolism, hepcidin regulation and erythropoiesis. Such evidence is essential to ensure that the benefits of LDA in preventing PE are not offset by unrecognised haematologic harms.

### Implications

**For clinicians:** Current evidence does not clearly establish whether LDA influences Hb or anaemia outcomes during pregnancy. For clinicians, this underscores the importance of interpreting LDA-related haematologic findings within the broader context of local anaemia prevalence, comorbidities and antenatal care practices.

**For researchers:** The review highlights substantial research gaps, particularly the absence of studies that systematically evaluate Hb or iron-related endpoints in pregnant women receiving LDA. Future work may benefit from incorporating standardised haematologic measures and exploring mechanistic pathways in diverse populations, including LMIC settings.

**For policymakers:** Policy frameworks may consider the need for more locally relevant evidence on anaemia and LDA use, especially in contexts where anaemia and infectious comorbidities are highly prevalent. Strengthening routine data systems and surveillance for both HDP and anaemia may help inform future guideline refinement.

### Limitations

As a scoping review, this work was designed to map the extent, nature and characteristics of the available evidence on LDA and anaemia-related outcomes in pregnancy, rather than to synthesise findings across studies, assess the risk of bias of individual studies or evaluate the certainty of evidence. Consequently, no formal quality appraisal, quantitative synthesis or assessment of causal effects was undertaken. In addition, the review may be subject to publication bias, selective outcome reporting and language bias, particularly given that anaemia-related outcomes were often not predefined endpoints in the included studies. The heterogeneity in study designs, populations, aspirin dosing, outcome definitions and reporting further limits direct comparison across studies.

Despite these limitations, this scoping review provides a descriptive overview of the existing evidence base and highlights critical gaps in the literature – particularly the paucity of pregnancy-specific data on Hb, iron status and anaemia outcomes in LMIC settings. These findings can inform the design of future primary studies and targeted systematic reviews aimed at evaluating the haematologic effects of LDA in pregnancy.

## Conclusion

Low-dose aspirin has transformed the landscape of PE prevention and is increasingly embedded in antenatal guidelines worldwide. Robust evidence from RCTs and systematic reviews demonstrates that its efficacy in reducing early-onset PE, without a consistent signal of major bleeding in predominantly well-nourished populations. However, its potential impact on Hb and anaemia risk during pregnancy remains underexplored.

The limited available data – comprising two pregnancy studies with Hb endpoints and several trials reporting bleeding outcomes – do not demonstrate a clear detrimental effect of LDA on maternal Hb concentration. At the same time, mechanistic studies suggest biologically plausible pathways through which LDA could influence iron balance, either adversely (through gastrointestinal blood loss or altered iron bioavailability) or beneficially (via anti-inflammatory and hepcidin-modulating effects). In LMICs, where anaemia, infection and nutritional deficiencies are common, these uncertainties acquire particular relevance. Low-dose aspirin use should be accompanied by careful attention to baseline and interval anaemia assessment, especially in women with comorbid disease or high inflammatory burden. Targeted research in LMIC populations – combining clinical, mechanistic and implementation perspectives – is required to ensure that the benefits of LDA are fully realised while monitoring for potential haematologic consequences in vulnerable groups.

## References

[1] Li F, Wang T, Chen L, Zhang S, Chen L, Qin J. Adverse pregnancy outcomes among mothers with hypertensive disorders in pregnancy: A meta-analysis of cohort studies. Pregnancy Hypertens. 2021;24:107–117. 10.1016/j.preghy.2021.03.001 33813363

[2] Poon LC, Shennan A, Hyett JA, et al. The International Federation of Gynecology and Obstetrics (FIGO) initiative on pre-eclampsia: A pragmatic guide for first-trimester screening and prevention. Int J Gynaecol Obstet. 2019;145 (Suppl 1):1–33. 10.1002/ijgo.12892 PMC6944283 31111484

[3] Jikamo B, Adefris M, Azale T, Alemu K. Incidence, trends and risk factors of preeclampsia in sub-Saharan Africa: A systematic review and meta-analysis. PAMJ-One Health. 2023;11:1. 10.11604/pamj-oh.2023.11.1.39297

[4] Noubiap JJ, Bigna JJ, Nyaga UF, et al. The burden of hypertensive disorders of pregnancy in Africa: A systematic review and meta-analysis. J Clin Hypertens. 2019;21(4):479–488. 10.1111/jch.13514 PMC8030504 30848083

[5] Moodley J, Soma-Pillay P, Buchmann E, Pattinson RC. Hypertensive disorders in pregnancy: 2019 national guideline [homepage on the Internet]. Pretoria: University of Pretoria; 2019 [cited 2025 Apr 14]. Available from: https://repository.up.ac.za/handle/2263/76064 31635598

[6] Committee on Obstetric Practice. ACOG committee opinion no. 743: Low-dose aspirin use during pregnancy. Obstet Gynecol. 2018;132(1):e44–e52. 10.1097/AOG.0000000000002580 29939940

[7] Rolnik DL, Wright D, Poon LC, et al. Aspirin versus placebo in pregnancies at high risk for preterm preeclampsia. N Engl J Med. 2017;377(7):613–622. 10.1056/NEJMoa1704559 28657417

[8] South African National Department of Health. Guidelines for maternity care in South Africa, 2016 [homepage on the Internet]. Pretoria: NDoH; 2016 [cited 2025 Nov 17]. Available from: https://knowledgehub.health.gov.za/system/files/elib_cache/16/93/1693462755/Guidelines_for_Maternity_Care_in_South_Africa_2016.pdf

[9] Jena MK, Sharma NR, Petitt M, Maulik D, Nayak NR. Pathogenesis of preeclampsia and therapeutic approaches targeting the placenta. Biomolecules. 2020;10(6):953. 10.3390/biom10060953 32599856 PMC7357118

[10] Redman CWG, Sargent IL. Immunology of pre-eclampsia. Am J Reprod Immunol. 2010;63(6):534–543. 10.1111/j.1600-0897.2010.00831.x 20331588

[11] Patrono C, García Rodríguez LA, Landolfi R, Baigent C. Low-dose aspirin for the prevention of atherothrombosis. N Engl J Med. 2005;353(22):2373–2383. 10.1056/NEJMra052717 16319386

[12] Walsh S. Low-dose aspirin: Treatment for the imbalance of increased thromboxane and decreased prostacyclin in preeclampsia. Am J Perinatol. 1989;6(2):124–132. 10.1055/s-2007-999562 2653334

[13] World Health Organization. Anaemia [homepage on the Internet]. Geneva: WHO; 2023 [cited 2025 Jun 20]. Available from: https://www.who.int/news-room/fact-sheets/detail/anaemia

[14] Kassa GM, Muche AA, Berhe AK, Fekadu GA. Prevalence and determinants of anemia among pregnant women in Ethiopia; a systematic review and meta-analysis. BMC Hematol. 2017;17(1):17. 10.1186/s12878-017-0090-z 29075500 PMC5646153

[15] Silubonde TM, Smuts CM, Ware LJ, Chidumwa G, Malan L, Norris SA. Determinants of anaemia among women of reproductive age in South Africa: A Healthy Life Trajectories Initiative (HeLTI). PLoS One. 2023;18(3):e0283645. 10.1371/journal.pone.0283645 36996088 PMC10062540

[16] Chu FC, Shao SSW, Lo LM, Hung TH. Association between maternal anemia at admission for delivery and adverse perinatal outcomes. J Chin Med Assoc. 2020;83(4):402–407. 10.1097/JCMA.0000000000000215 32238782 PMC13048132

[17] Kabir MA, Rahman MM, Khan MN. Maternal anemia and risk of adverse maternal health and birth outcomes in Bangladesh: A nationwide population-based survey. PLoS One. 2022;17(12):e0277654. 10.1371/journal.pone.0277654 36525409 PMC9757595

[18] Young MF, Oaks BM, Tandon S, Martorell R, Dewey KG, Wendt AS. Maternal hemoglobin concentrations across pregnancy and maternal and child health: A systematic review and meta-analysis. Ann NY Acad Sci. 2019;1450(1):47–68. 10.1111/nyas.14093 30994929 PMC6767572

[19] Dewey KG, Oaks BM. U-shaped curve for risk associated with maternal hemoglobin, iron status, or iron supplementation. Am J Clin Nutr. 2017;106:1694S–1702S. 10.3945/ajcn.117.156075 29070565 PMC5701708

[20] Jirakittidul P, Sirichotiyakul S, Ruengorn C, Techatraisak K, Wiriyasirivaj B. Effect of iron supplementation during early pregnancy on the development of gestational hypertension and pre-eclampsia. Arch Gynecol Obstet. 2018;298(3):545–550. 10.1007/s00404-018-4821-6 29951711

[21] Mtali YS, Lyimo MA, Luzzatto L, Massawe SN. Hypertensive disorders of pregnancy are associated with an inflammatory state: Evidence from hematological findings and cytokine levels. BMC Pregnancy Childbirth. 2019;19(1):237. 10.1186/s12884-019-2383-7 31288789 PMC6617701

[22] Scholten RR, Lotgering FK, Hopman MT, et al. Low plasma volume in normotensive formerly preeclamptic women predisposes to hypertension. Hypertension. 2015;66(5):1066–1072. 10.1161/HYPERTENSIONAHA.115.05934 26370891

[23] Torres HRM, Gonzalez JB, Honorato RR. Microangiopathic hemolytic anemia associated with preeclampsia. Int J Biomed Res Prac. 2022;2(1):1–4. 10.33425/2769-6294.1009

[24] Peters MDJ, Marnie C, Tricco AC, et al. Updated methodological guidance for the conduct of scoping reviews. JBI Evid Synth. 2020;18(10):2119–2126. 10.11124/JBIES-20-00167 33038124

[25] Tricco AC, Lillie E, Zarin W, et al. PRISMA Extension for Scoping Reviews (PRISMA-ScR): Checklist and explanation. Ann Intern Med. 2018;169(7):467–473. 10.7326/M18-0850 30178033

[26] McGowan J, Sampson M, Salzwedel DM, Cogo E, Foerster V, Lefebvre C. PRESS peer review of electronic search strategies: 2015 guideline statement. J Clin Epidemiol. 2016;75:40–46. 10.1016/j.jclinepi.2016.01.021 27005575

[27] Rayyan. Rayyan: Faster systematic reviews [homepage on the Internet]. 2022 [cited 2023 Sept 23]. Available from: https://www.rayyan.ai/

[28] Levac D, Colquhoun H, O’Brien KK. Scoping studies: Advancing the methodology. Implement Sci. 2010;5:69. 10.1186/1748-5908-5-69 20854677 PMC2954944

[29] Page MJ, McKenzie JE, Bossuyt PM, et al. The PRISMA 2020 statement: An updated guideline for reporting systematic reviews. Br Med J. 2021;372:n71. 10.1136/bmj.n71 33782057 PMC8005924

[30] Jessani S, Saleem S, Hoffman M, et al. Association of haemoglobin levels in the first trimester and at 26–30 weeks with fetal and neonatal outcomes: A secondary analysis of the global network for women’s and children’s health’s ASPIRIN trial. BJOG. 2021;128(9):1487–1496. 10.1111/1471-0528.16676 33629490 PMC8286300

[31] Ngcobo NM, Dorsamy V, Bagwandeen C, Mkhize PZ, Moodley J. The impact of low-dose aspirin on hemoglobin levels in pregnancy: A secondary analysis of a randomized controlled trial for prevention of hypertensive disorders of pregnancy. Int J Gynaecol Obstet. 2025. 10.1002/ijgo.70710 PMC13094701 41321324

[32] Plancha M, Brito ME, Ferreira LC, Oliveira N. Life-threatening upper gastrointestinal bleeding in a pregnant woman under low-dose aspirin – Case report and literature review. Perinat J. 2024;32(1):66–69. 10.59215/prn.24.0321011

[33] Hastie R, Tong S, Wikström AK, Sandström A, Hesselman S, Bergman L. Aspirin use during pregnancy and the risk of bleeding complications: A Swedish population-based cohort study. Am J Obstet Gynecol. 2021;224(1):95.e1–95.e12. 10.1016/j.ajog.2020.07.023 32687818

[34] White KJ, Son M, Lundsberg LS, et al. Low-dose aspirin during pregnancy and postpartum bleeding. Am J Perinatol. 2023;40(13):1390–1397. 10.1055/a-2096-5199 37211010

[35] Souter V, Painter I, Sitcov K, Khalil A. Propensity score analysis of low-dose aspirin and bleeding complications in pregnancy. Ultrasound Obstet Gynecol. 2024;63(1):81–87. 10.1002/uog.27472 37674400

[36] Lin L, Huai J, Li B, et al. A randomized controlled trial of low-dose aspirin for the prevention of preeclampsia in women at high risk in China. Am J Obstet Gynecol. 2022;226(2):251.e1–251.e9. 10.1016/j.ajog.2021.08.004 34389292

[37] Zhang F, Wang H. Effect of low-dose aspirin intervention on pre-eclampsia prevention in high-risk pregnant women and its impact on postpartum hemorrhage. Front Med (Lausanne). 2024;11:1414697. 10.3389/fmed.2024.1414697 39526246 PMC11543432

[38] Jiang Y, Chen Z, Chen Y, et al. Low-dose aspirin use during pregnancy may be a potential risk for postpartum hemorrhage and increased blood loss: A systematic review and meta-analysis. Am J Obstet Gynecol MFM. 2023;5(4):100878. 10.1016/j.ajogmf.2023.100878 36706919

[39] Roberge S, Bujold E, Nicolaides KH. Aspirin for the prevention of preterm and term preeclampsia: Systematic review and meta-analysis. Am J Obstet Gynecol. 2018;218(3):287–293.e1. 10.1016/j.ajog.2017.11.561 29138036

[40] Henderson JT, Whitlock EP, O’Connor E, Senger CA, Thompson JH, Rowland MG. Low-dose aspirin for prevention of morbidity and mortality from preeclampsia: A systematic evidence review for the US Preventive Services Task Force. Ann Intern Med. 2014;160(10):695–703. 10.7326/M13-2844 24711050

[41] Duley L, Meher S, Hunter KE, Seidler AL, Askie LM. Antiplatelet agents for preventing pre-eclampsia and its complications. Cochrane database Syst Rev. 2019;10:CD004659. 10.1002/14651858.CD004659.pub3 PMC6820858 31684684

[42] US Preventive Services Task Force, Davidson KW, Barry MJ, et al. Aspirin use to prevent preeclampsia and related morbidity and mortality: US Preventive Services Task Force recommendation statement. J Am Med Assoc. 2021;326(12):1186–1191. 10.1001/jama.2021.14781 34581729

[43] Balarajan Y, Ramakrishnan U, Özaltin E, Shankar AH, Subramanian SV. Anaemia in low-income and middle-income countries. Lancet. 2011;378(9809):2123–2135. 10.1016/S0140-6736(10)62304-5 21813172

[44] Kontoghiorghes GJ. The puzzle of aspirin and iron deficiency: The vital missing link of the iron-chelating metabolites. Int J Mol Sci. 2024;25(10): 5150. 10.3390/ijms25105150 38791185 PMC11121054

[45] Kontoghiorghes GJ. Drug selection and posology, optimal therapies and risk/benefit assessment in medicine: The paradigm of iron-chelating drugs. Int J Mol Sci. 2023;24(23):16749. 10.3390/ijms242316749 38069073 PMC10706143

[46] Xiao S, Guo L, Zhang M, Hu R, Liu R. Low-dose aspirin may prevent preeclampsia by inhibiting the expression of ATF2. Endocr Metab Immune Disord Drugs Targets. 2023;23(5):702–710. 10.2174/1871530323666221103105349 36330631

[47] Obeagu EI, Obeagu GU, Ukibe NR, Oyebadejo SA. Anemia, iron, and HIV: Decoding the interconnected pathways: A review. Medicine. 2024;103(2):e36937. 10.1097/MD.0000000000036937 38215133 PMC10783375

[48] Cao G, Wang Y, Wu Y, Jing W, Liu J, Liu M. Prevalence of anemia among people living with HIV: A systematic review and meta-analysis. eClinicalMedicine. 2022;44:101283. 10.1016/j.eclinm.2022.101283 35128369 PMC8803600

[49] Mokhele I, Jinga N, Berhanu R, Dlamini T, Long L, Evans D. Treatment and pregnancy outcomes of pregnant women exposed to second-line anti-tuberculosis drugs in South Africa. BMC Pregnancy Childbirth. 2021;21:453. 10.1186/s12884-021-03956-6 34182944 PMC8240388

[50] Bauserman M, Leuba SI, Hemingway-Foday J, et al. The efficacy of low-dose aspirin in pregnancy among women in malaria-endemic countries. BMC Pregnancy Childbirth. 2022;22(1):303. 10.1186/s12884-022-04652-9 35399060 PMC8994890

[51] Abioye AI, Fawzi WW. Nutritional anemias. In: Present knowledge in nutrition [homepage on the Internet]. Elsevier; 2020 [cited 2025 May 22], p. 503–521. Available from: https://www.sciencedirect.com/science/article/pii/B9780128184608000277

[52] Darnton-Hill I, Mkparu UC. Micronutrients in pregnancy in low-and middle-income countries. Nutrients. 2015;7(3):1744–1768. 10.3390/nu7031744 25763532 PMC4377879

[53] Kumar A, Bhattacharya S. Sickle cell disease: A comparative perspective on global and national initiatives. Front Hematol. 2024;3:1457158. 10.3389/frhem.2024.1457158

[54] Arese P, Gallo V, Pantaleo A, Turrini F. Life and death of glucose-6-phosphate dehydrogenase (G6PD) deficient erythrocytes–role of redox stress and band 3 modifications. Transfus Med Hemother. 2012;39(5):328–334. 10.1159/000343123 23801924 PMC3678266

[55] King SE. Relations between antenatal quality of care and iron folic acid adherence: A multi-country study [homepage on the Internet]. [PhD Thesis]. Johns Hopkins University; 2022 [cited 2025 May 22]. Available from: https://jscholarship.library.jhu.edu/bitstream/handle/1774.2/67486/KING-DISSERTATION-2022.pdf?sequence=1

[56] Galloway R, McGuire J. Determinants of compliance with iron supplementation: Supplies, side effects, or psychology? Soc Sci Med. 1994;39(3):381–390. 10.1016/0277-9536(94)90135-X 7939855

